# Antibody-Independent Control of γ-Herpesvirus Latency via B Cell Induction of Anti-Viral T Cell Responses

**DOI:** 10.1371/journal.ppat.0020058

**Published:** 2006-06-23

**Authors:** Kelly B McClellan, Shivaprakash Gangappa, Samuel H Speck, Herbert W. Virgin

**Affiliations:** 1 Department of Pathology and Immunology, Washington University School of Medicine, Saint Louis, Missouri, United States of America; 2 Emory Transplant Center, Emory University School of Medicine, Atlanta, Georgia, United States of America; 3 Division of Microbiology and Immunology, Yerkes Regional Primate Research Center, Emory University, Atlanta, Georgia, United States of America; Robarts Research Institute, Canada

## Abstract

B cells can use antibody-dependent mechanisms to control latent viral infections. It is unknown whether this represents the sole function of B cells during chronic viral infection. We report here that hen egg lysozyme (HEL)-specific B cells can contribute to the control of murine γ-herpesvirus 68 (γHV68) latency without producing anti-viral antibody. HEL-specific B cells normalized defects in T cell numbers and proliferation observed in B cell−/− mice during the early phase of γHV68 latency. HEL-specific B cells also reversed defects in CD8 and CD4 T cell cytokine production observed in B cell−/− mice, generating CD8 and CD4 T cells necessary for control of latency. Furthermore, HEL-specific B cells were able to present virally encoded antigen to CD8 T cells. Therefore, B cells have antibody independent functions, including antigen presentation, that are important for control of γ-herpesvirus latency. Exploitation of this property of B cells may allow enhanced vaccine responses to chronic virus infection.

## Synopsis

B cells can control virus infection by making specific antibodies that bind to virus and infected cells. However, it is unknown whether B cells perform other anti-viral functions to protect the host during infection. The authors addressed this question by infecting mice with murine γ-herpesvirus 68 (γHV68), a relative of Epstein-Barr virus and Kaposi's sarcoma associated virus, which establishes lifelong latent infection in mice. Mice lacking B cells (B cell−/−) failed to control latent γHV68 infection and had reduced T cell activity compared to wild-type mice. In order to determine if virus non-specific B cells can control latency, the authors engineered mice that contain B cells, but cannot generate antibodies that bind to γHV68. Virus non-specific B cells restored normal control of latency and T cell activation defects observed in B cell−/− mice, and could present virus-encoded antigen to T cells. Thus, B cells can play a critical role in control of chronic viral infection independent of their capacity to make anti-viral antibody. Defining the mechanisms for this unique activity of B cells may provide novel ways to treat or control chronic viral infection.

## Introduction

γ-Herpesviruses such as Epstein Barr virus (EBV), Kaposi's sarcoma herpesvirus (KSHV), and murine γ–herpesvirus 68 (γHV68) latently infect lymphocytes and other cells as part of a strategy for maintaining life-long infection. Latent infection represents a balance between the virus and the host to which immunity makes an essential contribution. γ-herpesvirus latency and replication of virus that has reactivated from latently infected cells contribute to γ-herpesvirus-associated diseases [1–7]. The stability of this balance between virus and host is demonstrated by the observation in mice that a latency “set point” exists such that the same number of cells are latently infected regardless of the dose or route of infection [8], and in humans by the observation that individuals have a stable level of EBV latency over years [9]. Despite the stability of γ-herpesvirus latency, the balance between virus and host is delicate since γ-herpesvirus-induced disease is most often seen in immunocompromised hosts. In addition, deletion of individual host [7,10,11] or viral [12–14] genes disrupts this balance with consequent inefficient infection or development of disease.

To understand the stable but delicate balance between the host and γ-herpesviruses present during life-long infection, it is necessary to define mechanisms of immunity responsible for holding the virus at bay. To define these mechanisms many groups have studied infection of mice with γHV68, which provides a relevant small animal model for γ-herpesvirus infection and immunity. After clearance of acute infection, γHV68 latently infects macrophages, B cells, and dendritic cells [8,15–18]. γHV68 infection is associated with development of B cell malignancies, vasculitis, and atherosclerosis [2,7,19,20].

Immunity controls latent γHV68 infection by limiting the number of cells carrying viral genome during latency [10,21,22] and by regulating the efficiency with which these cells reactivate from latency when explanted [10,11,23,24]. In addition, the immune system regulates persistent viral replication, which is detected as the presence of preformed infectious virus in tissues after clearance of the acute infection [7,10,11,17,23]. Persistent γHV68 replication is distinct from replication occurring during acute infection (acute replication) since the γHV68 v-cyclin and v-Bcl-2 genes are required for persistent but not acute replication [12–14]. Persistent replication is observed in normal mice, and is more prominent in immunocompromised mice such as those lacking B cells or interferon-γ (IFNγ) [7,10,14,17,23]. It is likely that persistent replication involves virus that has reactivated from latently infected cells since the v-cyclin and v-Bcl-2 genes are required for both efficient reactivation from latency and for persistent replication [12,13]. Persistent replication may contribute to latency via infection of new cells that enter the latent pool [25,26].

There are two forms of γHV68 latency that are distinguishable experimentally [8,10–12]. The early form of latency is measurable 16 d after infection when acute infection has been cleared. At this time most cells carrying latent viral genome reactivate when cultured ex vivo [11]. The late form of latency, typically measured at 42 d after infection, is characterized by inefficient reactivation ex vivo with 10% or less of genome bearing cells reactivating when explanted [10,11]. Latency is typically measured in the spleen as a lymphoid site and the peritoneum as a body cavity site. Analysis of these sites is of interest since both EBV and KSHV establish latency in lymphoid sites and KSHV causes body cavity based lymphomas [3–5]. Both the early and late forms of latency in splenocytes and peritoneal cells are observed regardless of the route (intraperitoneal or intranasal) or dose of viral inoculation [8].

Several components of the immune system contribute to the control of latent and persistent γHV68 infection. These include CD8 T cells [10,21,22], CD4 T cells [21,27–29], perforin [10], granzymes [30], caspase 3 [30], IFNγ [6,7,10,24,29], IFNαβ [23], and antibody and B cells [11,25,26]. Of these, the role of B cells is of particular interest because γ-herpesviruses including EBV, KSHV, and γHV68 establish latency in B cells. In addition to serving as a site of latent infection, B cells regulate latency in non-B cells [11]. B cell−/− mice exhibit increased frequencies of reactivating and viral genome bearing cells compared to B6 mice [11], and show increased persistent replication in lung and aorta [6,7,14,31]. Thus, removal of one latent reservoir, B cells, increases the level of latency in non-B cells, and diminishes control of persistent replication. Lack of specific anti-viral antibody likely explains some of the abnormalities in latency and persistence seen in B cell−/− mice. Passive transfer of antibody significantly decreases the frequency of latently infected cells in B cell−/− or T cell depleted CD28−/− mice [25,26]. However, it is not known whether the important role of B cells in control of γHV68 latency and persistent replication is explained by production of anti-viral antibody or other antigen-specific B cell receptor (BCR)-dependent activities of B cells.

To identify B cell activities during infection that are independent of antibody production and expression of a virus antigen-specific BCR we bred a mouse that contained B cells but cannot mount an antigen specific anti-viral B cell response (HELMET mice). We found that the abnormal control of latency in B cell−/− mice was largely normalized by the presence of viral antigen non-specific B cells. Furthermore, abnormalities in CD8 and CD4 T cell expansion and IFNγ production observed in B cell−/− mice were reversed in HELMET mice. Depletion of either T cell subset resulted in increased latent infection in HELMET mice but had minimal effects in B cell−/− mice. B cells therefore have critically important antibody- and antigen-specific BCR-independent functions during chronic γHV68 infection that are at least in part explained by B cell-dependent induction of CD8 and CD4 T cell responses.

## Results

### HEL-Specific B Cells Decrease the Frequency of Splenocytes That Reactivate from Latency and Decrease the Efficiency of Reactivation from Latency

**Figure 1:**
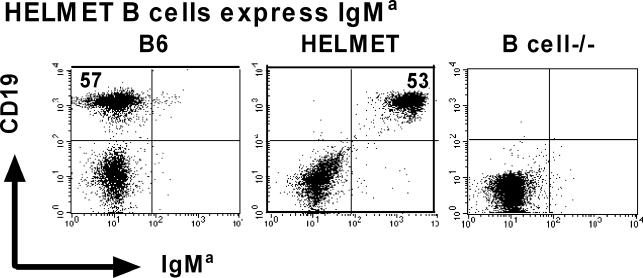
HELMET Mice Contain Transgenic B Cells Splenocytes from naïve B6, HELMET, and B cell−/− mice were stained with antibodies against CD19 and IgM^a^. Numbers represent percentages of cells in the indicated quadrants.

B cell−/− mice have higher numbers of cells carrying latent γHV68 than wild type mice [11]. We wanted to determine whether the effects of B cells on latent γHV68 infection require production of anti-viral antibody and engagement of a virus antigen-specific BCR. One approach to this question would be to study the effects of adoptively transferred B cells on γHV68 infection of B cell−/− mice. However, adoptively transferred B cells do not persist in this setting [32,33], necessitating a genetic approach to engrafting virus antigen non-specific mice onto a B cell−/− background. We therefore bred the HEL-specific IgM/IgD BCR bearing the IgM^a^ allotype from MD4 mice [34] onto the μMT B cell−/− background [35] (Figure 1, HELMET mice). HELMET mice contained IgM^a^ and CD19 double positive B cells in spleen (Figure 1) and lymph nodes (unpublished data) while B6 mice and B cell−/− mice did not.

**Figure 2:**
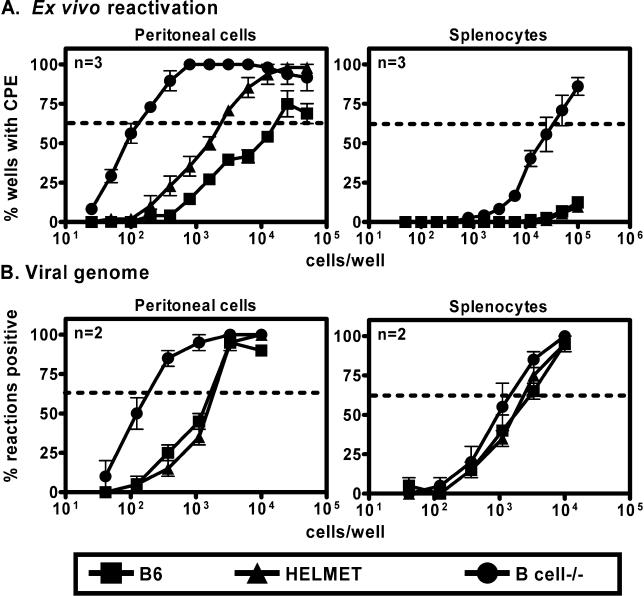
Virus Non-Specific B Cells Control Latent Infection (A) Ex vivo reactivation from latency in peritoneal cells (left) and splenocytes (right) from B6, HELMET, and B cell−/− mice 42 d after infection. Dotted lines indicate the 63.2% Poisson distribution line used to calculate the frequency of cells reactivating virus. (B) Frequency of cells from peritoneal cells (left) and splenocytes (right) carrying γHV68 genome in B6, HELMET, and B cell−/− mice at day 42 post infection. Dotted lines indicate the 63.2% Poisson distribution line used to calculate the frequency of cells carrying viral genome. As indicated in the Materials and Methods section, results from 10–15 total mice from two to three independent experiments ± SEM are reported here.

To determine if HEL-specific B cells can control the late form of latency in a lymphoid site we examined splenic latency in B6, HELMET, and B cell−/− mice (Figure 2). We confirmed a prior report [11] that the frequency of splenocytes from B cell−/− mice that reactivate from latency ex vivo is significantly elevated compared to the frequency observed in splenocytes from B6 mice (*p* = 0.003, Figure 2A). The frequency of splenocytes from HELMET mice that reactivated ex vivo was indistinguishable from the frequency observed in splenocytes from B6 mice and was significantly lower than that observed in splenocytes from B cell−/− mice (*p* = 0.0001). Therefore, the presence of HEL-specific B cells normalized defects in the control of splenic latency observed in B cell−/− mice.

We next determined whether effects of HEL-specific B cells on splenic latency were due to alterations in the frequency of viral genome bearing cells or in the efficiency of reactivation of explanted latently infected cells (defined as the frequency of cells reactivating ex vivo divided by the frequency of cells bearing viral genome multiplied by 100). The frequencies of genome positive splenocytes in B cell−/−, HELMET, and B6 mice were similar (Figure 2B, right panel). About 15% of viral genome positive splenocytes from B cell−/− mice reactivated ex vivo compared to about 2% in B6 mice and 3% in HELMET mice (Figures 2A and 2B, right panels). Therefore the effects of HEL-specific B cells on splenic latency were due to changes in the efficiency of ex vivo reactivation rather than the frequency of cells bearing viral genome.

### HEL-Specific B Cells Decrease the Frequency of Peritoneal Cells That Reactivate from Latency and That Carry Latent γHV68 Genome

To determine whether viral antigen non-specific B cells can restore normal regulation of latency in a body cavity site we compared the late form of latency in B6, HELMET, and B cell−/− mice. Consistent with previous results [11], the frequency of reactivating cells in B cell−/− mice was 200-fold higher than in B6 mice (Figure 2A, left panel, *p* = 0.008). HEL-specific B cells were able to decrease reactivation from latency in peritoneal cells since the frequency of reactivating cells was 20-fold less in HELMET mice in comparison to B cell−/− mice (*p* = 0.0053). However, the frequency of reactivating peritoneal cells in HELMET mice was 11-fold higher than that in peritoneal cells from B6 mice (Figure 2A, left panel, *p* = 0.05). These data demonstrate that HEL-specific B cells limit reactivation from latency in peritoneal cells, but do not fully compensate for the presence of normal B cells.

We next determined whether the decreased frequency of peritoneal cells that reactivate from latency in HELMET mice compared to B cell−/− mice was due to a decrease in the frequency of latently infected cells by measuring the efficiency of reactivation. Consistent with published results [11], the frequency of viral genome-positive cells was 10-fold higher in peritoneal cells from B cell−/− mice than in B6 mice (Figure 2B, left panel, *p* = 0.01). Thus, roughly 100% of latently infected peritoneal cells from B cell−/− mice reactivated from latency when explanted while only 6% of latently infected peritoneal cells from B6 mice reactivated (compare Figures 2A and 2B). HEL-specific B cells decreased the frequency of cells carrying latent viral genome to levels similar to those observed for B6 mice (*p* = 0.36). However, about 81% of viral genome bearing peritoneal cells from HELMET mice reactivated when explanted. Thus the effect of HEL-specific B cells on latency in peritoneal cells is largely via decreasing the frequency of cells carrying latent viral genome.

### The Effects of HEL-Specific B Cells on γHV68 Latency Are Not Due to Cross-Reactivity between HEL and γHV68

**Figure 3:**
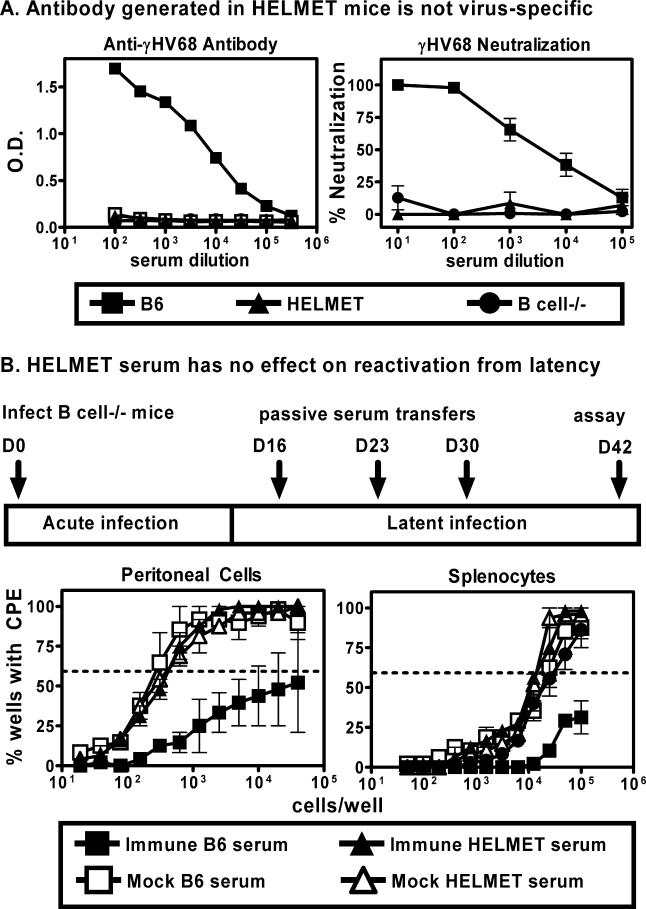
Control of γHV68 Latency by HEL-Specific B Cells Is Not due to Production of Cross-Reactive Antibody (A) (Left) ELISA for γHV68-specific antibodies in serum from mock infected and virus infected B6, HELMET, and B cell−/− mice. Data are representative of two independent experiments. (Right) Sera from mock infected and γHV68 infected B6, HELMET, and B cell−/− mice were incubated with γHV68 and tested for their ability to neutralize γHV68 infection. Percent neutralization was normalized to results obtained using serum from mock-infected mice of the same genotype. (B) Beginning at day 16 post-infection, B cell−/− mice were treated with serum from mock infected or γHV68 infected B6 and HELMET mice. Ex vivo reactivation from latency was assessed 42 d after infection.

The data provided above show that the presence of HEL-specific B cells has significant effects on γHV68 latency in two different sites. To determine whether these effects were virus-specific antibody independent we needed to determine whether anti-γHV68 antibody is generated in either B cell−/− or HELMET mice. To address this issue we used enzyme-linked immunosorbent assay (ELISA), viral neutralization assays, and passive transfer experiments (Figure 3).

As expected, anti-viral antibody was found by ELISA in serum from infected B6 mice but not in serum from infected B cell−/− or HELMET mice (Figure 3A, left panel). Antibody specific to HEL was detected in mock infected and γHV68 infected HELMET mice (unpublished data). Similarly, serum from γHV68 infected B6 mice neutralized γHV68, while serum from infected B cell−/− or HELMET mice did not (Figure 3A, right panel). Thus anti-HEL antibodies do not cross-react with γHV68 in ELISA or neutralization assays and infected B cell−/− and HELMET mice fail to make detectable antibody to γHV68.

To rule out the possibility that ELISA and neutralization assays failed to detect physiologically relevant anti-γHV68 antibody in the serum of HELMET mice and that HEL specific antibody in the serum of HELMET mice might alter γHV68 latency, we compared the effect of passively transferred serum from infected HELMET or B6 mice on latency in B cell−/− mice. We and others previously demonstrated that the passive transfer of anti-viral antibody can decrease the number of latently infected cells [25] and decrease the recrudescence of lytic virus [26] in latently infected mice by blocking lytic viral replication [25]. The transfer of control serum from either B6 or HELMET mice had no effect on latency in B cell−/− mice (compare Figures 2A and 3B). As previously published [25], passively transferred immune serum from γHV68 infected B6 mice decreased the frequency of peritoneal cells reactivating from latency more than 100-fold compared to the transfer of control serum from mock infected B6 mice (Figure 3B, *p* = 0.05), and of splenocytes more than 4-fold (Figure 3B, *p* = 0.01). In contrast, serum from infected HELMET mice had no effect on latency (Figure 3B).

Together these data show that there is no γHV68-reactive-antibody in infected HELMET mice and that HEL-specific antibody in serum of HELMET mice did not alter γHV68 latency. We conclude that the control of latency observed in HELMET mice (Figures 2A and 2B) is dependent on the presence of HEL-specific B cells rather than antibody or a virus antigen-specific BCR.

### Effects of HEL-Specific B Cells on Latency Are Not Explained by Decreases in Acute γHV68 Replication

**Figure 4:**
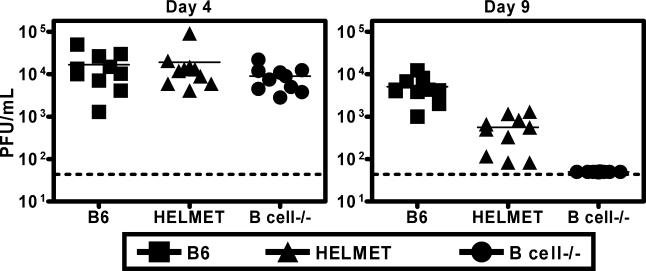
HEL-Specific B Cells Restore Acute Phase γHV68 Replication to the B Cell−/− Background Splenic viral titer measured at days 4 and 9 after γHV68 infection. Solid lines represent the mean viral titer. Dotted lines represent the limit of detection of the assay. Each symbol represents the viral titer in a single mouse. Data were pooled from two independent experiments.

The above data showed that B cells that do not produce anti-viral antibody or express a virus antigen-specific BCR can nevertheless have significant effects on latent γHV68 infection. To define the mechanism responsible for this effect we first determined whether effects of HEL-specific B cells on acute γHV68 replication might explain decreases in γHV68 latency detected in HELMET mice. We therefore measured γHV68 replication in B6, HELMET, and B cell−/− mice 4 and 9 d after infection. 4 d after infection, viral titers were similar in B6, HELMET, and B cell−/− mice (*p* > 0.2, Figure 4). On day 9, γHV68 titers were more than 100-fold greater in B6 mice (*p* < 0.0001) than in B cell−/− mice (Figure 4), reflecting the importance of B cells for maintaining γHV68 replication [36]. The presence of HEL-specific B cells led to increased γHV68 replication compared to B cell−/− mice (*p* < 0.0001) 9 d after infection, but did not fully normalize splenic titers (*p* < 0.0001). These data show that decreased latency in HELMET mice compared to B cell−/− mice cannot be explained by decreased γHV68 replication in HELMET mice.

### HEL-Specific B Cells Correct Abnormalities in Splenocyte Numbers Observed in B cell−/− Mice

**Figure 5:**
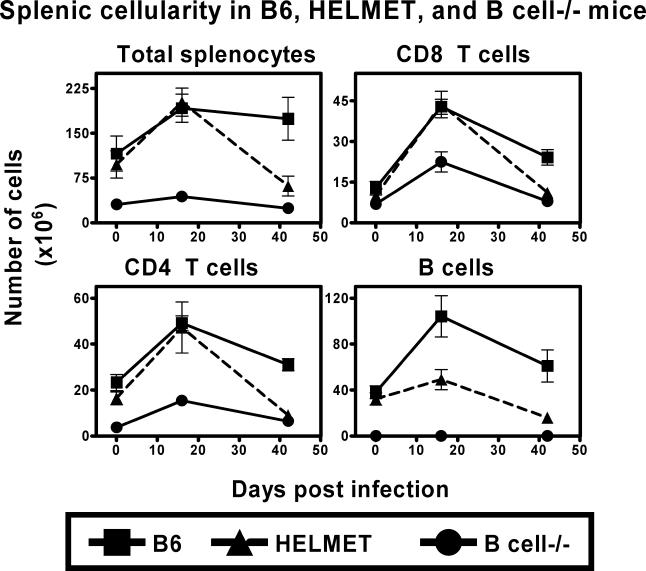
HEL-Specific B Cells Normalize Some but Not All Abnormalities in Splenic Cellularity Observed in B Cell−/− Mice Splenocytes from pooled naïve or mock infected and γHV68 infected B6, HELMET, and B cell−/− mice were counted and stained with antibodies against CD8, CD4, and CD19. The splenocyte numbers were the same in naïve and mock-infected mice, and data from mock infected and naïve mice were pooled. Numbers represent number of cells/spleen × 10^6^. The data are pooled from two to six independent experiments evaluating three to five mice per experiment.

Based on studies in other systems comparing responses to infection in the presence and absence of B cells [33,37–40], we reasoned that increases in T cell responses in the presence of HEL-specific B cells might contribute to control of γHV68 latency. We therefore determined whether HEL-specific B cells restore abnormalities in splenic cellularity that have been reported in B cell−/− mice including reduced numbers of splenocytes, CD8 T cells, and CD4 T cells compared to B6 mice [41]. We confirmed that mock infected or naïve B cell−/− had decreased numbers of CD4 T cells compared to B6 mice [41] (Figure 5). In contrast to B cell−/− mice, we found that the absolute number of splenocytes, B cells, CD4 T cells, and CD8 T cells was comparable between in naïve and/or mock-infected B6 and HELMET mice (Figure 5). Thus, the presence of HEL-specific B cells effectively corrected deficiencies in T cell numbers found in uninfected B cell−/− mice.

We next evaluated the T and B cell response to γHV68 infection in HELMET mice compared to B6 and B cell−/− mice by quantifying the spleen cell populations 16 and 42 d after infection. Consistent with previous data [36], we found that γHV68 infection of B6 increased total splenocytes (*p* = 0.03), CD4 T cells (*p* = 0.03), CD8 T cells (*p* < 0.0001), and B cells (*p* = 0.02) compared to uninfected mice 16 d after infection. B cell−/− mice did not show increased total splenocytes (*p* = 0.37) or CD4 T cells (*p* > 0.05). Although there were slight increases in CD8 T cells (*p* = 0.005) in B cell−/− mice, the number was significantly reduced in comparison to the number of CD8 T cells in B6 mice 16 d after infection (*p* = 0.0036). At 16 d after infection the presence of HEL-specific B cells normalized total splenocyte numbers (*p* = 0.78) and CD4 (*p* = 0.91), and CD8 T cell numbers (*p* = 0.81) to levels observed in B6 mice (Figure 5). Taking these data together, HEL-specific B cells restored defects in cell numbers observed in infected B cell−/− mice 16 d after infection. We did not identify all of the types of cells present in the spleens of the different mice used here, and thus it is possible that the presence of B cells alters the number of cells other than CD4 and CD8 T cells. The B cell response to infection observed in B6 mice was not fully restored by the presence of HEL-specific B cells (Figure 5). In addition, HEL-specific B cells did not restore CD4 or CD8 responses to the level seen in B6 mice 42 d after infection. Thus, HEL-specific B cells can normalize splenic lymphocyte numbers in uninfected mice early after infection with γHV68, but HEL-specific B cells do not replace all normal B cell functions.

### HEL-Specific B Cells Are Activated during Early γHV68 Latency

**Figure 6:**
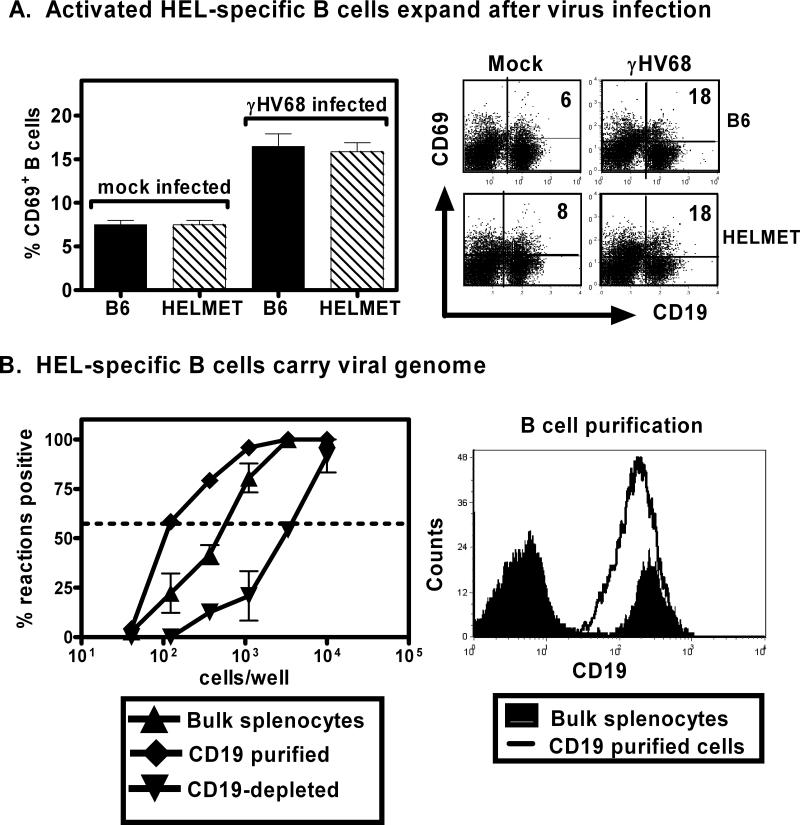
HEL-Specific B Cells Are Activated during γHV68 Infection and Are Infected by γHV68 (A) Splenocytes from mock infected and γHV68 infected B6 and HELMET mice were stained with antibodies specific for CD19 and CD69 16 d after infection. Shown are the percentages of splenic CD19^+^ cells that express CD69 (left). Shown are representative dot plots (right). (B) Frequency splenic HEL-specific B cells that carry viral genome 16 d post infection (left). Shown is a representative histogram of the purity of the B cell population after purification (right). These data are representative of two independent experiments using five mice per experiment.

The normalization of T cell proliferative responses early after infection in the presence of HEL-specific B cells raised the question of whether HEL-specific B cells are activated in response to infection. To address this we used flow cytometry to determine the percentage of activated B cells in B6 and HELMET mice 16 d after infection (Figure 6). Compared to mock-infected controls, we found that the percentage of B cells that expressed CD69 increased 2-fold in both B6 (*p* = 0.01) and HELMET mice (*p* = 0.002) (Figure 6A) compared to mock infected mice. Further histological examination analysis showed that germinal center-like structures formed in HELMET and B6 mice after γHV68 infection (unpublished data). Thus, HEL-specific B cells can be activated during γHV68 infection independent of a virus antigen-specific BCR or antiviral antibody.

We questioned whether this activation might be explained by direct infection of HEL-specific B cells by γHV68. We therefore determined whether HEL specific B cells carry latent γHV68 16 d after infection. We found that approximately 1/150 CD19+ cells in HELMET mice carried viral genome compared to 1/700 total splenocytes (Figure 6B). We entertained the possibility that our purification procedure non-specifically enriched for viral genome bearing cells, but found that <1:10,000 CD8 T cells purified in parallel, carried viral genome (unpublished data). As an additional control we determined the frequency of viral genome bearing cells in CD19-depleted splenocytes. We found that only 1:4,400 CD19-depleted splenocytes carried viral genome. By multiplying these frequencies times the numbers of cells per spleen from Figure 5, we determined that HEL-specific B cells account for the majority of latently infected splenocytes in HELMET mice (3.3 × 10^5^ viral genome bearing B compared to 3.4 × 10^4^ viral genome bearing non-B cells). These findings in HELMET mice agree with previously reported data in B6 mice showing that B cells carry the majority of γHV68 genome 16 d after infection [17,18,42]. Interestingly, the frequency of viral genome bearing HEL-specific B cells was 33-fold lower than the frequency of HEL-specific B cells expressing CD69 (compare Figures 6A and 6B), indicating that direct virus infection cannot fully explain activation of HEL-specific B cells during γHV68 infection.

### Virus Non-Specific B Cells Rescue T Cell Defects in B Cell−/− Mice

Taken together the experiments above indicate that HEL-specific B cells play an important role in generating a full CD4 and CD8 T cell proliferative response to γHV68 infection. Since CD4 and CD8 T cells are important for the control of γHV68 latency [10,21,22,27], we postulated that HEL-specific B cells could control latent γHV68 infection by promoting protective CD4 and CD8 T cell responses. To address this postulate, we further characterized T cell expansion and activation in B6, HELMET, and B cell−/− mice after infection.

**Figure 7:**
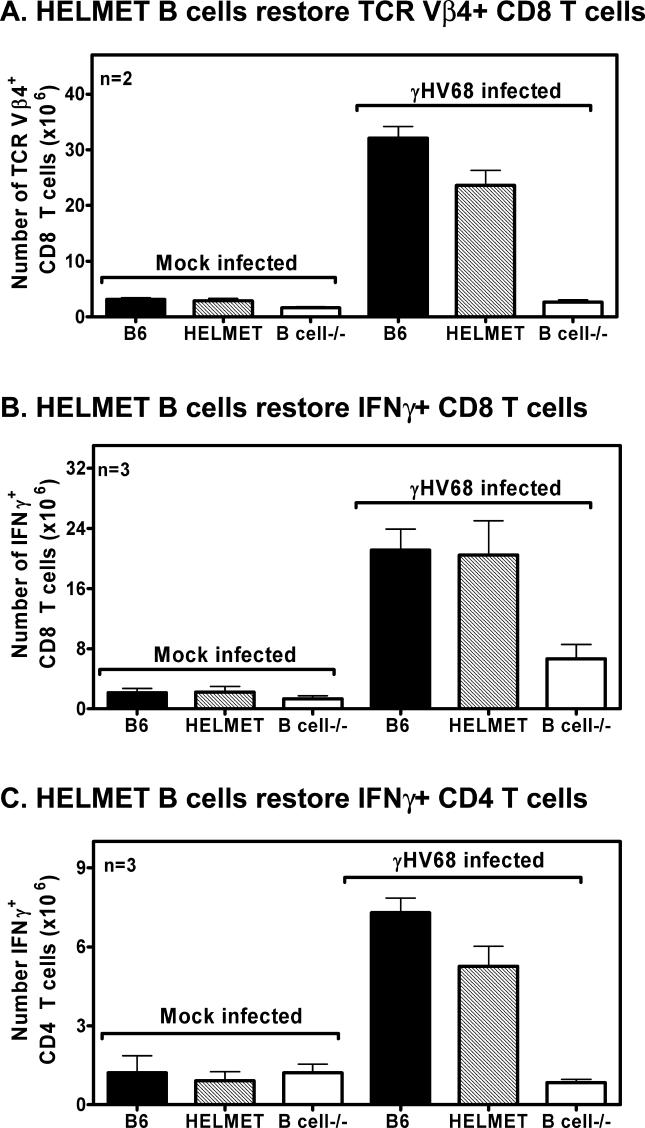
Virus Non-Specific B Cells Restore CD8 T Cell Defects Found in B Cell−/− Mice Splenocytes from mock infected and γHV68 infected B6, HELMET, and B cell−/− mice at day 21 (A) or day 16 (B and C) were counted and stained with antibodies specific for CD8 and TCR Vβ4 (A) or CD8, CD4, and IFNγ (B and C). Shown are the numbers of CD8 T cells/spleen that are TCR Vβ4^+^ (A) and the numbers of CD8 and CD4 T cells/spleen that produce IFNγ upon restimulation with PMA/ionomycin (B and C). These data were pooled from two to three independent experiments using three to five mice per experiment.

The CD8 T cell response to γHV68 is heavily biased toward T cells whose receptors are encoded by the Vβ4 gene, and expansion of Vβ4+ CD8 T cells is dependent on the presence of B cells [43–46]. We used flow cytometry to evaluate the Vβ4^+^ T cell receptor (TCR)+ CD8 T cell expansion at 21 d after infection, a time when it has been consistently observed in wild-type mice [47] (Figure 7). Flow-cytometry revealed a 10-fold expansion of Vβ4^+^TCR+ CD8 T cells in B6 (*p* = 0.0053) and HELMET mice (*p* = 0.016) relative to mock-infected controls (Figure 7A). As expected, we did not detect the Vβ4^+^TCR+ CD8 T cell expansion after γHV68 infection in B cell−/− mice. Thus, the B cell-dependent induction of Vβ4+ CD8 T cell responses to γHV68 does not required anti-viral antibody or a virus antigen-specific BCR.

To further examine T cell activation, we used flow cytometry to assess the number of CD8 and CD4 T cells that produce IFNγ. Chronic activation of CD8 T cells has been reported during long term γHV68 infection [48–50]. We measured the number of T cells that produce IFNγ 16 d after infection based on studies showing that day 16 falls within the peak of T cell activation during γHV68 infection [50] and that IFNγ is important for the control of γHV68 latency [10,24]. Compared to mock infection, γHV68 at day 16 induced a 10-fold expansion of CD8 T cells that produced IFNγ in B6 (*p* = 0.0027) and HELMET mice (*p* = 0.01) while no significant expansion was observed in B cell−/− mice (Figure 7B). Additionally, γHV68 infection induced a significant expansion of CD8 T cells that expressed CD69 in B6 and HELMET mice while no significant expansion was found in B cell−/− mice (unpublished data). Similar studies of CD4 T cells revealed a 6-fold expansion of IFNγ producing CD4 T cells in B6 mice (*p* = 0.002) and in HELMET mice (*p* = 0.006), but not in B cell−/− mice (Figure 7C). Together these data show that the presence of HEL-specific B cells allows the induction of an activated population of CD8 and CD4 T cells in γHV68 infected mice.

### HELMET Mice Use Both CD4 T Cell- and CD8 T Cell-Dependent Mechanisms to Limit γHV68 Latent Infection

**Figure 8:**
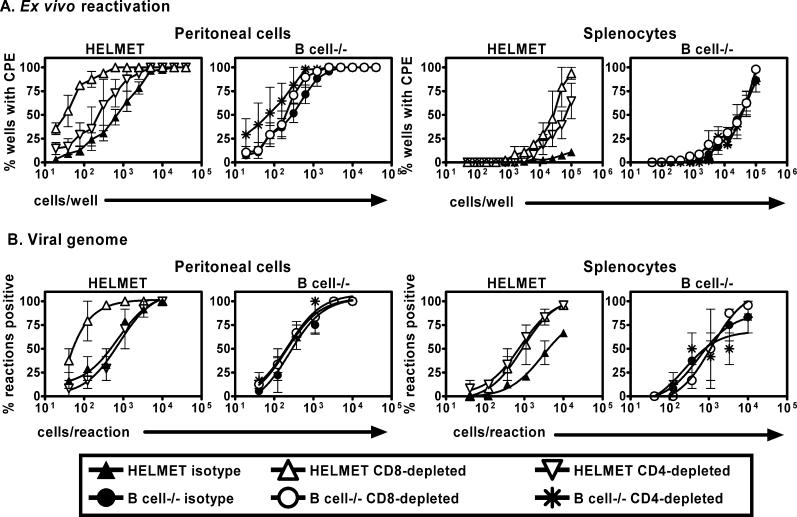
CD8 T Cells Induced by the Presence of HEL-Specific B Cells Are Essential for Control of γHV68 Latency (A) Effects of CD8- and CD4-depletion on the frequency of cells reactivating ex vivo from latency in peritoneal cells (left two graphs) and splenocytes (right two graphs) 42 d after infection in HELMET and B cell−/− mice. (B) Effects of CD8- and CD4-depletion on the frequency of cells carrying viral genome in peritoneal cells and splenocytes 42 d after infection. These data were pooled from two to three independent experiments using three to five mice per experiment.

We questioned whether the observed differences between HELMET and B cell−/− mice in T cell numbers and activation were functionally relevant to the control of latency. We reasoned that activated T cells present in HELMET mice and wild-type mice but not B cell−/− mice might contribute to the control of latent γHV68 infection observed in B6 and HELMET mice. To investigate this, we determined the effect of T cell depletion on latency in B cell−/− and HELMET mice (Figure 8).

Treatment with isotype control had some effect on levels of peritoneal cell latency in both B cell−/− and HELMET mice (compare Figures 2A and 8A). We therefore considered the levels of latency observed in isotype control antibody treated mice as the proper control for treatment with T cell depleting antibodies. CD4-depletion of B cell−/− mice had a small effect on latency in peritoneal cells relative to isotype-control treated mice (Figure 8A), increasing the frequency of reactivating peritoneal cells 3-fold (*p* < 0.0001) in both HELMET mice and B cell−/− mice. CD4-depletion had no effect on the frequency of viral genome bearing peritoneal cells in HELMET mice or in B cell−/− mice compared to isotype control treated mice. Thus, CD4-depletion increased the efficiency with which peritoneal cells reactivate from latency.

We observed that CD4-depletion had no effect on latency in splenocytes from B cell−/− mice (Figure 8A and 8B). However, we observed a five-fold increase in reactivating HELMET splenocytes upon CD4-depletion (*p* < 0.0001, Figure 8A). CD4-depletion also increased the frequency of splenocytes carrying viral genome more than 6-fold in HELMET mice (*p* < 0.0001, Figure 8B). Therefore, the increase in reactivation was partially due to an increase in the frequency of viral genome-bearing splenocytes. We interpreted these data to mean that CD4 T cells are important for the control of latent γHV68 infection in HELMET mice.

As previously shown [29], CD8 T cell depletion of B cell−/− mice had no effect on the frequency of peritoneal cells that reactivated ex vivo or the frequency of viral genome-bearing peritoneal cells compared to mice treated with isotype-control antibodies (Figures 8A and 8B). Depletion of CD8 T cells from HELMET mice resulted in a 15-fold increase in the frequency peritoneal cells that reactivated from latency compared to isotype-control treated groups (*p* < 0.0001, Figure 8A). This increase in reactivation was largely due to an increase in the frequency of cells harboring viral genome because 12-fold more peritoneal cells carried viral genome in CD8 T cell-depleted HELMET mice compared to isotype control treated HELMET mice (*p* < 0.0001, compare Figures 8A and 8B). Thus, CD8 T cells are important for controlling latency in peritoneal cells from HELMET mice.

Similar to the results obtained from peritoneal cells, CD8-depletion of B cell−/− mice had no effect on the frequency of splenocytes reactivating ex vivo or the frequency of viral genome-bearing splenocytes compared to isotype-control treated mice (Figures 8A and 8B). Administration of isotype control antibodies had no effect on splenic latency (compare Figures 2A and 8A). However, the frequency of splenocytes reactivating ex vivo in CD8 T cell depleted-HELMET mice was conservatively estimated to be at least 3-fold greater than in isotype control treated HELMET mice (*p* < 0.0001, Figure 8A). Furthermore, CD8-depletion led to a 5-fold increase in the frequency of splenocytes carrying viral genome compared to isotype control treated HELMET mice (*p* < 0.0001 Figure 8B).

Together these data indicate that CD4 and CD8 T cell dependent mechanisms control γHV68 latency in HELMET mice, but not in B cell−/− mice.

### Virus Non-Specific B Cells Present Virally Encoded Antigen to CD8 T Cells

**Figure 9:**
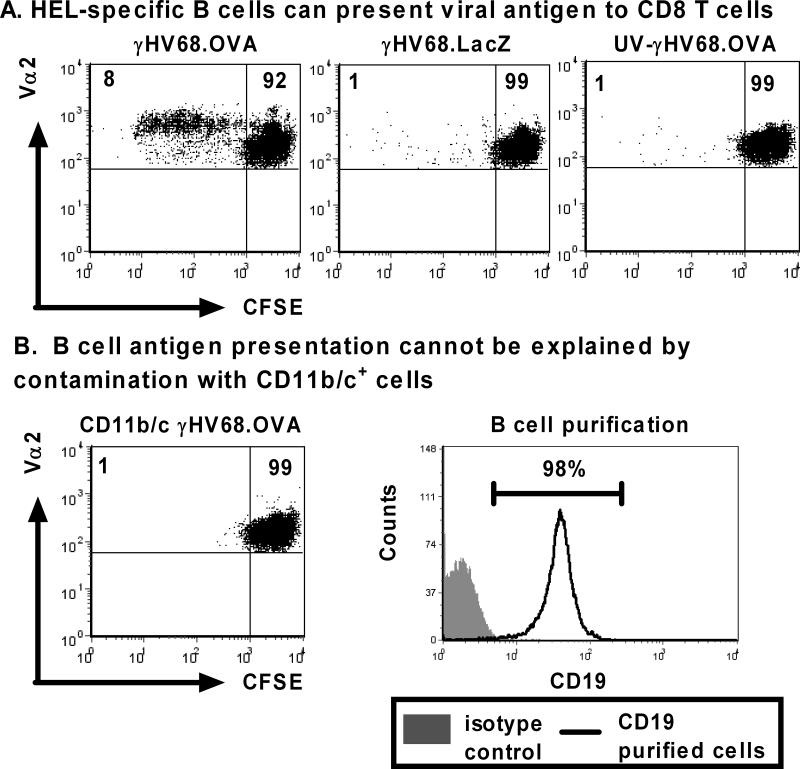
Virus Non-Specific B Cells Present γHV68-Encoded Ag to CD8 T Cells (A) Antigen presentation by HEL-specific B cells infected with γHV68.OVA, γHV68.LacZ, or UV-inactivated γHV68.OVA. Purified B cells were incubated with CFSE labeled OT1 T cells to determine their ability to present antigen. Antigen presentation was measured by the ability of APCs to induce proliferation in OT1 T cells, as shown by dilution of CFSE after cell division. Shown are representative dot plots from flow cytometry experiments with percentages noted in the quadrants. Plots were gated on TCR Vα2^+^ cells. (B) Contamination of purified B cells with CD11b/c^+^ cells cannot account antigen presentation (left panel). Purity of fractionated CD19^+^ cells (right panel). These data are representative of two to six independent experiments using three to five mice per experiment.

We hypothesized that antigen presentation by B cells could provide one explanation for the observed differences in CD8 T cell activation and function between HELMET and B cell−/− mice. To test this hypothesis, antigen presentation assays were performed using the response of T cell receptor transgenic T cells specific for the SIINFEKL epitope of ovalbumin (OVA, OT1 T cells, Figure 9). Purified splenic B cells from HELMET mice were infected in vitro with either γHV68.OVA or γHV68.LacZ and subsequently cultured with CFSE labeled OT1 CD8 T cells. We quantitatively measured the ability of infected B cells to induce antigen-specific OT1 T cell proliferation. γHV68.OVA infected B cells were able to induce about 10-fold more OT1 T cell proliferation than γHV68.LacZ infected B cells (*p* = 0.03, Figure 9A). We additionally found that antigen presentation was dependent on the number of antigen presenting cells (APCs) (unpublished data). To determine whether virus replication was required for antigen presentation, we incubated B cells with UV-inactivated γHV68.OVA. Incubation with UV-inactivated γHV68.OVA did not induce OT1 proliferation beyond levels observed with γHV68.LacZ infection (Figure 9A). Therefore, effective antigen presentation required virus replication.

Although the fractionated B cell population was >98% pure across multiple experiments (Figure 9B) and we depleted CD11b/c+ cells prior to B cell purification, we wanted to exclude the possibility that the < 2% contaminating cells (< 1 × 10^4^ cells) accounted for the observed antigen presentation. To address this issue, antigen presentation assays were performed using purified CD11b/c+ cells in place of B cells. While purified CD11b/c+ cells were able to elicit OT1 proliferation at higher numbers (unpublished data), < 1 × 10^4^ purified CD11b/c+ cells were unable to induce significant OT1 proliferation (Figure 9B), indicating that contaminating CD11b/c+ cells in purified B cell preparations did not explain antigen presentation by infected B cells. Taking these data together, we conclude that B cells present virally encoded antigen to CD8 T cells.

## Discussion

We show here that B cells can have a significant role in the control of chronic γ-herpesvirus infection even when they do not produce antiviral antibody and do not express a virus antigen-specific B cell receptor. The presence of virus-antigen non-specific B cells normalized defective CD4 and CD8 T cell responses observed in B cell−/− mice, generating T cells sufficient for the control of latent infection. Furthermore, B cell activation during γ-herpesvirus infection occurs independent of virus antigen-specific BCR expression, a phenomenon that is not explained by latent infection of proliferating B cells. Identification of physiologically important effects of virus antigen non-specific B cells on virus infection suggests that exploitation of this property of B cells may allow enhanced vaccine responses to chronic virus infection. Identification of molecular mechanisms responsible for these antibody- and BCR-independent effects on virus infection, and for B cell activation during virus infection in the absence of cognate antigen, may provide fundamental insights into B cell biology and immune physiology.

### Viral Antigen Non-Specific B Cells Can Control Chronic γHV68 Infection

Since B cell−/− mice lack B cells as well as antiviral antibody, it was unclear whether phenotypes observed in these mice such as increased γHV68 latency and increased persistent γHV68 replication are due to the lack of B cells, the lack of antiviral antibody, or both [7,11,14,31]. Previous reports found that a significant portion of the polyclonal antibody response that occurs after γHV68 infection is not specific to γHV68 [25,50,51], suggesting that virus antigen non-specific B cell responses are induced by γHV68 infection. The physiologic importance of these responses was not clear, but provided an impetus for development of the HELMET mouse model for analysis of virus antigen non-specific B cells during γHV68 infection.

We conclude here that B cells can control both the number of latently infected cells and the efficiency with which these cells reactivate from latency without producing antibody or being activated by a virus-antigen specific BCR. The lack of a complete cellular and molecular definition of γHV68 latency led us to measure latency using the simple parameters of the number and reactivation phenotype of the total latent cell pool. Thus, these studies demonstrate a fundamentally important function of antigen non-specific B cells on the overall level of latency, but do not address the role of B cells in the regulation of latency in individual cell compartments or at the molecular level.

Our conclusion that virus non-specific B cells can control latent γHV68 infection rests in part on our data showing that HEL-specific antibody does not bind γHV68 specifically as measured by neutralization assays or ELISA. However, since not all physiologically important activities of antibody are recapitulated in in vitro assays [52–54], we also performed passive transfer experiments to test for the presence of serum components that would control γHV68 infection. Despite the capacity of immune serum from normal mice to control infection in B cell−/− mice, serum from HELMET mice had no effect on latent infection. These data conclusively show that production of virus-specific antibody is not required in order for B cells to have profound effects on chronic herpesvirus infection.

### Antibody-Dependent Effects of B Cells on γHV68 Infection

While we show here that not all effects of B cells on chronic γHV68 infection require antiviral antibody production, previously published data and data presented here make it clear that antigen specific B cell responses play an important role in control of γHV68 infection and the normal immune response to γHV68 infection. Passive transfer of polyclonal antibody or antibody specific for lytic cycle viral antigens significantly decreases acute and latent γHV68 infection [25,26,55]. Furthermore, passive transfer of immune serum prior to infection can significantly limit latent infection [56]. Antibody specific for γHV68 can neutralize virus infectivity (this study and [25,50]). Together these data convincingly show that specific antibody can limit γHV68 infection.

In addition to studies directly showing that antibody specific for γHV68 can control γHV68 infection, data from this study shows that HEL-specific B cells fail to fully complement all abnormalities observed in γHV68 infected B cell−/− mice. For example, the presence of HEL-specific B cells failed to reconstitute abnormal splenic T cell responses in B cell−/− mice observed 42 d after γHV68 infection (Figure 5), and did not completely normalize latency in peritoneal cells. This may reflect a need for either antibody or normal B cell differentiation driven by antigen specific BCR stimulation in maintenance of T cell responses during chronic γHV68 infection. Furthermore, γHV68 establishes latency in a memory B cell compartment [15–18], further suggesting that normal B cell differentiation is important for aspects of γHV68 pathogenesis and latency. All of these data support the concept that antigen specific B cell responses are important during γHV68 infection both by provision of immune antibody and via roles of normal B cell differentiation in viral infection.

### Mechanisms by Which HEL-Specific B Cells Contribute to Control of Latent Infection

There are multiple explanations for how HEL-specific B cells might control latency, but we focused our attention on the possibility that the mechanism of control of latency in HELMET mice involved B cell dependent T cell responses. Other groups have found that CD8 T cell responses to several pathogens including lymphocytic choriomeningitis virus [39] and Listeria monocytogenes [38] are abnormal in B cell−/− mice. We therefore addressed two points: (i) whether T cell responses to γHV68 in B cell−/− mice are abnormal during chronic γHV68 infection, and if so (ii) whether HEL-specific B cells are able to normalize T cell responses. We confirmed prior reports that the expansion of Vβ4+ CD8 T cells induced by γHV68 infection is compromised in B cell−/− mice [43,44,46,57]. While the physiologic role of these Vβ4^+^ CD8 T cells has not been demonstrated [58], their expansion provides one measure of CD8 T cell proliferative responses to γHV68 infection. Abnormal Vβ4 biased CD8 responses observed in B cell−/− mice were restored by the presence of HEL-specific B cells. Several investigators have observed chronic activation of T cells during γHV68 infection [48,49]. We found that both CD4 and CD8 T cells were activated in γHV68 infected B6 and HELMET mice, but not in B cell−/− mice.

These results, together with the demonstration that CD4 and CD8 T cells are critical for control of latent γHV68 infection [10,21,22,27], led us to question whether the correlation between T cell expansion and activation and control of γHV68 latency in HELMET mice was physiologically meaningful. Similar to previous reports [29], CD8 T cell depletion had no effect on latent infection in B cell−/− mice. However, CD4 T cell depletion slightly increased the frequency of reactivating peritoneal cells in B cell−/− mice. Depletion of either T cell subset increased latency in HELMET mice. We interpreted these data to mean that CD4 and CD8 T cells were functionally relevant to the control of γHV68 latency in HELMET mice. Thus, the presence of HEL-specific B cells resulted in expansion and activation of CD4 and CD8 T cells that are required for control of γHV68 infection.

Since B cells are the major reservoir of γHV68 latency in the spleen of normal mice [18,42,58], the latency observed in B cell−/− mice resides in non-B cells. HEL-specific B cells decreased splenic latency overall, despite being themselves latently infected (Figures 2A and 2B). Thus, the presence of virus antigen non-specific B cells is associated with significant decreases in splenic latency in non-B cells. Our data do not directly address whether B cell-dependent T cells control latency in B cells. Additionally, while we showed that HEL-specific B cell control viral latency in splenocytes and peritoneal cells, our data do not address whether this effect is direct or via control of the persistent replication in the lung or aorta that have been observed in B cell−/− mice [7, 17,59]. As the level of latency in B cell−/− mice is dependent on ongoing replication of γHV68 [25], it is possible that T cells control latency by controlling persistent replication at sites other than the spleen or peritoneum.

### How Do HEL-Specific B Cells Promote Normal T Cell Responses?

Several groups have shown that antigen non-specific B cells can take up and present LCMV-derived peptides [60] and HEL-derived peptides [61] to CD4 T cells. In addition, γHV68 infected B cells can present a ligand to CD8 T cells bearing Vβ4 with consequent activation of the Vβ4^+^ T cells [46,57]. These data, together with the demonstration that HEL-specific B cells can be infected by γHV68, suggest the possibility that HEL-specific B cells may function as antigen presenting cells. Our data further support this hypothesis by demonstrating that HEL-specific B cells can present virally encoded antigen to CD8 T cells.

An alternative mechanism by which HEL-specific B cells might contribute to normalized T cell responses is via restoration of abnormalities in splenic architecture and chemokine environment observed in B cell−/− mice [41]. Intact splenic architecture is critical for the control of LCMV [62]. In this regard it is important to note that HEL-specific B cells restored some of the abnormalities in splenic cellularity observed in uninfected and infected B cell−/− mice. γHV68 infection of LTα−/− mice, which lack lymph nodes and have a disrupted splenic architecture [63], results in the eventual control of latent infection [64]. These data imply that, although intact splenic architecture is required for the control of some viral infections, it might not be an absolute requirement for controlling γHV68 latency. Data presented here do not rule out the possibility that intact splenic architecture in HELMET mice is important for T cell-mediated control of γHV68 latency.

### Activation of B Cells during γ-Herpesvirus Infection

During EBV infection, viral genes expressed in latently infected B lymphocytes [4] drive B cell expansion. In addition to proliferating, EBV transformed B cells express surface phenotypes consistent with those seen during antigen activation [65]. Studies of B cell activation during γHV68 infection have been limited to date. A component of the expansion of B cells in vivo during γHV68 is dependent on CD4 T cells [66]. However, exposure of splenocytes to γHV68 infection in culture results in a CD4 T cell independent expansion and activation of B cells to express CD69 [66,67]. The requirement for virus infection and BCR specificity in those studies was not established. However, data presented here argues that B cells can be activated in vivo independent of either antibody or a γHV68-specific BCR and that this is not explained by latent virus infection as might have been predicted based on studies with EBV.

It is notable that despite expressing CD69, HEL-specific B cells did not significantly proliferate during γHV68 infection. One explanation for this is that by fixing BCR expression at the IgM/IgD stage via transgenesis, we have prevented a physiologically important proliferative response that required cognate antigen-specific BCR engagement. Lastly, it may be that antibody produced by virus-specific B cells is critical to maintaining B cell responses, possibly through retaining and sequestering antigen.

### Implications of these Findings for Understanding of Immune Control of Chronic Viral Infection

It is not clear how general our finding that antigen non-specific B cells can influence anti-viral responses will be. It is possible, for example, that the tropism of γHV68 for B cells during both acute and chronic infection results in a fundamental role for antigen-non-specific B cells during this infection that will not be present in infections by other pathogens. However, several other studies have found a lack of correlation between anti-viral antibody effects and B cell effects on viral infection [39,68,69]. Together with studies presented here, these data suggest that virus antigen non-specific B cells may play a generally important role in the control of virus infection.

## Materials and Methods

### Mice, infections, cells, and tissue harvests.

HELMET mice were bred and maintained at Washington University School of Medicine, St. Louis, Missouri, United States, in accordance with all federal and University policies. B6 mice were purchased from The Jackson Laboratory, Bar Harbor, Maine, United States. Unless otherwise stated, mice were age and sex matched and infected at 7–12 wk of age with 1 × 10^6^ plaque forming units (PFU) of γHV68 by intraperitoneal injection (IP) in 500 μl complete DMEM [13]. Peritoneal cells, splenocytes, and lymph node cells were harvested and processed as described from groups of five mice per experiment in two to six independent experiments [36]. Crossing MD4 transgenic mice [34] on to the B6.129S2-*Igh-6^tm1Cgn^* (also referred to as μMT, B cell−/−) background generated HELMET mice and B cell−/− littermate controls used in these studies. The presence of HEL-specific B cells was confirmed by flow cytometric analysis of peripheral blood using allotype-specific antibodies to IgM^a^ (DS-1, Pharmingen, San Jose, California, United States).

### Viruses, tissue culture, neutralization, and plaque assays.

γHV68 clone WUMS (ATCC VR1465) was passaged, grown and titered in NIH 3T12 cells or BALB/c or C57Bl/6 (B6) murine embryonic fibroblasts (MEFs) as described [13]. γHV68.OVA and γHV68.LacZ were generated and handled as previously described [28]. Neutralization and plaque assays were performed as previously described [25].

### Latency assays.

The frequency of cells reactivating from latency ex vivo was determined using a limiting dilution reactivation assay as previously described [11]. Briefly, we performed serial 2-fold dilutions of harvested cells (24 wells per dilution starting at 1 × 10^5^ cells per well for splenocytes and 4 × 10^4^ cells per well for peritoneal cells). These cells were plated onto permissive mouse embryonic fibroblast monolayers for 21 d and then scored for cytopathic effect due to reactivating virus [11]. Preformed virus in tissues was detected by plating parallel cell samples that had been subjected to mechanical disruption using a procedure that does not significantly inactivate preformed virus [36]. Samples reported here did not contain detectable persistently replicating virus. The frequency of cells harboring the γHV68 genome was determined in serial dilutions of latently infected cells by a limiting dilution nested PCR assay that amplifies γHV68 gene 50 or gene 72 sequences with single copy sensitivity [11]. Ten PCR reactions were analyzed for each of six-cell dilutions per sample per experiment. There were no false-positive reactions in assays reported here, and all assays demonstrated approximately one-copy sensitivity for plasmid DNA, with control reactions containing 10, 1, or 0.1 copies of plasmid DNA positive in 87%, 37%, and 4% of all reactions.

### In vivo depletion of lymphocyte subsets and lymphocyte purification.

Monoclonal antibodies (mAbs) specific to CD8β, H35, [21] were used to deplete latently infected mice of CD8 T cells. Beginning 28 d after γHV68 infection, 1 mg of lymphocyte-depleting antibody or an isotype-matched control antibody, SFR3-DR5, IgG2b, ATCC HB-151, [70] was administered to each mouse by IP injection. mAb treatment was repeated every fourth day. The efficacy of depletion was monitored using flow cytometric analysis of splenocytes using anti-mouse CD8α. The efficacy of depletion was >95% in B cell−/− mice and >90% in HELMET mice. CD19-postive B cells and CD8^+^ T cells were fractionated to >99% purity via magnetic beads (Miltenyi Biotec, Auburn, California, United States) according to the manufacturer's directions for limiting dilution PCR assays.

For antigen presentation assays, APCs were isolated as previously described [71]. First, cells expressing CD11b and CD11c were isolated simultaneously using CD11b and CD11c microbeads (CD11b/c subset) (Miltenyi). CD19^+^ cells were subsequently isolated from the CD11b/c-depleted flow through. More than 84% of cells within the CD11b/c subset expressed CD11b, CD11c, or both CD11b and CD11c. In this population, about 10% of cells expressed both CD11b and CD19 and 6% expressed CD19 alone. Cells in the CD19^+^ subset were >98% CD19^+^. We did not detect any CD11c^+^ cells in the CD19^+^ fraction, however 4% of the CD19^+^ cells were also CD11b^+^. CD8 T cells were isolated from OT1 or OT1/RAG mice using magnetic beads according to the manufacturer's instructions (Miltenyi). CD8 T cells were then stained with CFSE using the Cell Trace™ CFSE proliferation kit according to the manufacturer's instructions (Molecular Probes, Eugene, Oregon, United States).

### Serum harvest, passive transfer, and ELISA.

Serum was collected from B6, HELMET, and B cell−/− mice 42 d after γHV68 (immune) or mock-virus stock (control) inoculation. Passive transfers were performed as previously described [25]. ELISAs were performed as previously described using paraformaldehyde fixed viral antigen and goat anti-mouse IgG (H+L) (Jackson ImmunoResearch, West Grove, Pennsylvania, United States) [25].

### Antibodies and flow cytometry.

The following antibodies and isotype-matched controls were purchased from BD Pharmingen (San Jose, California, United States): anti-CD4-FITC (RM4-5), anti-CD19-APC (1D3), anti-CD69-PE (H1.2F3), and unlabeled/purified anti-CD40 (HM40-3). Anti-CD8-TC (CT-CD8a) and anti-Vβ4-PE (CTVB4), anti-Vα2-APC (B20.1), and isotype-matched controls were purchased from Caltag (Burlingame, California, United States). Single-cell suspensions were incubated with Fc-block (2.4G2) and then stained for cell surface markers in 96-well round-bottom plates prior to analysis using a FACSCalibur cytometer BD Biosciences Pharmingen, and FCS Express, version 2 (De Novo software, Toronto, Ontario, Canada). For intracellular staining, cells were activated with 20ng/ml PMA plus1μM ionomycin for 4–6 h in the presence of GolgiPlug from the Cytofix/Cytoperm kit from BD Pharmingen. After staining the cells for surface markers, cells were fixed and permeabilized using the Cytofix/Cytoperm kit according to the manufacturer's instructions. Next, the cells were stained with antibodies specific to IFNγ, washed and resuspended in FACS buffer prior to analysis.

### Antigen presentation assays.

CD19^+^ and CD11b/c^+^ subsets were incubated with either γHV68.OVA or γHV68.LacZ [72] at a multiplicity of infection (MOI) of 10 for 1 h (positive and negative controls using 1nM SIINFEKL and media alone were also performed in each experiment). APCs were then washed and incubated with 2.5 × 10^5^ CFSE-labeled OT1 T cells in U-bottom 96-well plates with 200 μl of complete RPMI media with 1:3,500 dilution of anti-CD40 mAb. 4 d later, flow cytometry was performed to determine the percentage of proliferating T cells.

### Statistical methods.

All data were analyzed using GraphPad Prism software (GraphPad Software, San Diego, California, United States). Frequencies were obtained from the cell number at which 63% of the wells scored positive for either reactivating virus or the presence of viral genome based on Poisson distribution. Data were subjected to nonlinear regression analysis to obtain single-cell frequency for each limiting dilution analysis. The curves were statistically analyzed by unpaired *t* test. All differences not specifically stated to be significant were insignificant (*p* > 0.05). Acute virus titer data were analyzed with the nonparametric Mann-Whitney test. Data from experiments analyzed by flow cytometry were subjected to unpaired *t* tests. Except where noted, data shown are the mean of two to six independent experiments ± SEM.

## Abbreviations

γHV68: murine γ-herpesvirus 68
APC: antigen presenting cell
BCR: B cell receptor
EBV: Epstein-Barr virus
ELISA: enzyme-linked immunosorbent assay
HEL: hen egg lysozyme
IFNγ
interferon-γ;
KSHV: Kaposi's sarcoma associated virus
Vβ4+ TCR: variable region gene β4 containing T cell receptor
